# Endoscopic Balloon Dilation for Primary Obstructive Megaureter in Children: Early Outcomes and Complications—A Case Series

**DOI:** 10.3390/medicina61030479

**Published:** 2025-03-10

**Authors:** George Vlad Isac, Nicolae Sebastian Ionescu

**Affiliations:** 1 Department of Pediatric Surgery and Orthopedics, “Carol Davila” University of Medicine and Pharmacy, 050474 Bucharest, Romania; sebastian.ionescu@umfcd.ro; 2 Department of Pediatric Surgery, “M.S. Curie” Clinical Emergency Hospital for Children, 077120 Bucharest, Romania; 3 The Academy of Romanian Scientists, 50044 Bucharest, Romania; 4 Romanian Academy of Medical Sciences, 030167 Bucharest, Romania

**Keywords:** primary obstructive megaureter, endourologic treatment, high-pressure balloon dilation, double-J stent, complications

## Abstract

*Background and Objectives*: Congenital urological malformations are among the most frequent causes of pediatric chronic kidney disease. Endoscopic balloon dilation and ureteral stenting can be considered less invasive options compared to conventional surgery for primary obstructive megaureter (POM). Nevertheless, the long-term results and side effects of these methods have not yet been well documented. The purpose of this study is to analyze the effectiveness and safety of the endoscopic treatment of POM in children, with the aim of assisting clinical decision making and improving treatment plans. *Materials and Methods*: A retrospective longitudinal study was performed at the Pediatric Surgery Department of the “M.S. Curie” Emergency Clinical Hospital for Children in Bucharest between October 2020 and September 2024. Eleven endoscopic interventions were performed in five pediatric patients (four boys and one girl) who had six affected ureters, with a median age of 22 months. The inclusion criteria were retrovesical ureter dilation > 7 mm and no prior surgeries of the ureterovesical junction. Cases with secondary megaureters were excluded from the study. The procedures comprised HPEBD and temporary double-J (DJ) stent placement, with systematic postoperative monitoring. Success was defined as improvements in symptoms, a decrease in hydronephrosis, and the preservation of renal function. *Results*: A final success rate of 83.3% was achieved with endoscopic treatment. Complications were noted in 73% of cases: Clavien–Dindo Grade I (30%); Clavien–Dindo Grade II (20%); Clavien–Dindo Grade IIIb (50%). The documented complications consisted of balloon rupture, stent migration, restenosis, and febrile urinary tract infections (UTIs). Nonetheless, no major complications were observed. The postoperative monitoring showed that renal function was stable and that hydronephrosis had improved gradually. *Conclusions*: Endoscopic procedures offer a promising, minimally invasive treatment for POM in children with a good success rate. However, the high complication risk necessitates careful patient selection, post-surgery monitoring, and clear guidelines.

## 1. Introduction

Congenital urological malformations are the leading cause of chronic kidney failure in children and account for approximately one-quarter of the reasons for dialysis in adult patients. Dysplastic changes in the renal parenchyma may be present from birth in some cases. Urinary stasis can accelerate the progression to fibrosis and renal atrophy [1,2,3].

Primary obstructive megaureter (POM) is a rare congenital anomaly with an estimated incidence of 0.36 to 0.46 cases per 1000 live births [4]. The prevalence of POM varies significantly across different populations and age groups. Studies have shown a male predominance in the occurrence of POM, with a ratio of 7:1. This gender disparity suggests potential underlying genetic or developmental factors that predispose males to this condition more than females. In terms of age distribution, POM is often diagnosed in pediatric populations, particularly in infants and young children [5].

Although the exact etiology of obstructive megaureter remains unknown, the existence of a distal, juxtavesical, adynamic ureteral segment with ultrastructural changes disrupting smooth muscle contractility, and peristalsis is widely accepted. This may be associated with frank stenosis at the ureterovesical junction [1,6,7].

Until the early 2000s, the standard treatment was open ureteral reimplantation, a procedure associated with prolonged recovery and a high risk of postoperative bladder dysfunction. The last two decades have seen significant progress in therapeutic methods, with technological innovations and a better understanding of pathophysiological mechanisms. Advances in endourological technology have introduced minimally invasive alternatives, offering safer options for pediatric patients [8,9,10].

Most patients today benefit from conservative treatment. In cases requiring surgical treatment, the classical approach, represented by ureterovesical reimplantation, is complemented by minimally invasive methods. Endourological techniques such as high-pressure endoscopic balloon dilation and the placement of double-J stents initially used as temporary measures have proven effective and safe. These are associated with rapid recovery, reduced severe complications, and lower hospitalization costs. These techniques are also applicable to young children under one year of age, where ureteral reimplantation can be challenging and carries a risk of postoperative bladder dysfunction. However, specific anatomical and pathological features, such as the length of the stenotic ureteral segment and the characteristics of the ureteral orifice, influence the procedure’s success and complication rates [11,12,13,14].

Endoscopic balloon dilation has emerged as a prominent method in the management of primary obstructive megaureters. This procedure involves the insertion of a balloon catheter into the ureter, which is then inflated to dilate the obstructed segment. Studies have demonstrated that HPEBD is a safe and effective treatment option with good long-term outcomes. A series of cases showed that long-term restenosis occurred in only 12.2% of patients, and subsequent HEBD procedures were successful in 88.9% of these cases [15]. One of the primary advantages of the endoscopic approach is the preservation of the distal ureteral blood supply, which is often compromised during open surgery. This preservation reduces the risk of ischemic complications and promotes better healing outcomes [16].

However, the endoscopic approach is not without challenges. The procedure can be technically demanding, especially in smaller children, due to the limitations imposed by the size of the cystoscope and urethra. This can result in increased difficulty in instrument manipulation, potentially resulting in incomplete treatment or the need for additional interventions.

In terms of radiation exposure, patients undergoing endoscopic treatment are exposed to levels comparable to those used in endoscopic stone procedures. While this exposure is generally considered safe, it remains an important factor for consideration, especially in pediatric patients [17].

Complications associated with endoscopic treatment include infections related to the use of foreign materials, such as stents, and mechanical complications during the insertion and removal of these devices. Another critical aspect is the safety profile of the procedure. The complication rate for endoscopic treatment ranges between 40% and 70%, with potential risks including ureteral perforation and meatal avulsion [18].

Although high-pressure balloon endoscopic dilation and ureteral stenting have demonstrated efficacy in relieving ureterovesical junction obstruction in selected cases, long-term outcomes and the complication profile are not yet fully understood. This study aims to evaluate the effectiveness and safety profile of these endoscopic treatment modalities in children with primary obstructive megaureter. By comparing success rates, complications, and the need for further interventions, we aim to provide a comprehensive analysis in order to guide clinical decision making in managing this complex anomaly.

## 2. Material and Methods

### 2.1. Study Design

We initiated a retrospective longitudinal study in the Pediatric Surgery Department of the “M.S. Curie” Emergency Clinical Hospital for Children in Bucharest. The study analyzed pediatric patients diagnosed with primary obstructive megaureter who consecutively underwent endoscopic treatment at our institution from October 2020 to September 2024, tracking the incidents and complications associated with these procedures.

### 2.2. Diagnostic Evaluation (Figure 1 and Figure 2)

Primary obstructive megaureters were diagnosed based on imaging and clinical data according to the British Association of Paediatric Urologists’ (BAPU) recommendations [12].

**Figure 1:**
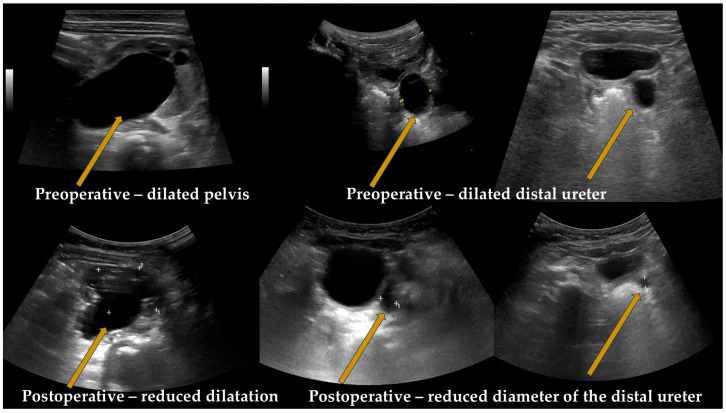
Ultrasound imaging.

**Figure 2:**
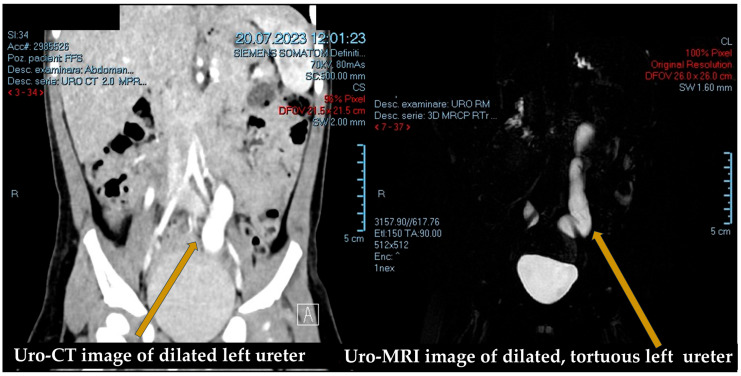
Uro-CT/Uro-MRI.

The imaging evaluation began with renal ultrasound (RUS), which provided detailed information regarding the renal parenchyma, renal pelvis, calyces, and distal ureter, allowing for an assessment of the degree of hydronephrosis and the presence of any associated renal anomalies (Figure 1).

Next, voiding cystourethrography (VCUG) was performed to rule out vesicoureteral reflux (VUR) and other lower urinary tract anomalies. Following this, uro-MRI or uro-CT was conducted (Figure 2). Magnetic resonance urography (MRU) is particularly effective in visualizing both the anatomy and function of the urinary tract. It accurately depicts the site and extent of ureteral obstruction, which is essential for planning appropriate treatment strategies. These imaging modalities also assess the degree of hydronephrosis and the presence of renal parenchymal damage, both of which are critical in determining the severity of obstruction and the urgency of intervention [8].

In cases where obstruction could not be demonstrated through other methods, diuretic renography using 99mTc-DTPA (diethylene-triamine-pentaacetate) was performed to evaluate renal perfusion, function, and drainage.

The severity of hydronephrosis was classified using the Society for Fetal Urology’s (SFU) grading system, which categorizes the condition into four grades based on sagittal ultrasound images [19].

### 2.3. Exclusion Criteria

Patients with obstructive–refluxive megaureter, ureteral duplication, ureterocele, neurogenic bladder, subvesical obstruction, or incomplete medical records were excluded. The selection criterion required the presence of a retrovesical ureter measuring > 7 mm in a patient with no prior surgeries at this level.

### 2.4. Surgical Indication

The surgical indications, based on the recommendations of the British Association of Paediatric Urologists, included progressive dilation associated with parenchymal thinning and impaired renal function (differential renal function [DRF] < 40% or a decline of >10% across successive examination, an obstructive pattern observed in the excretory phase of DTPA renal scintigraphy or recurrent febrile urinary tract infections) [1,13].

All patients underwent endoscopic balloon dilation with or without double-J stent placement as a primary intervention, with endourological treatment being the first-line therapeutic approach.

#### Surgical Procedure (Figure 3)

**Figure 3:**
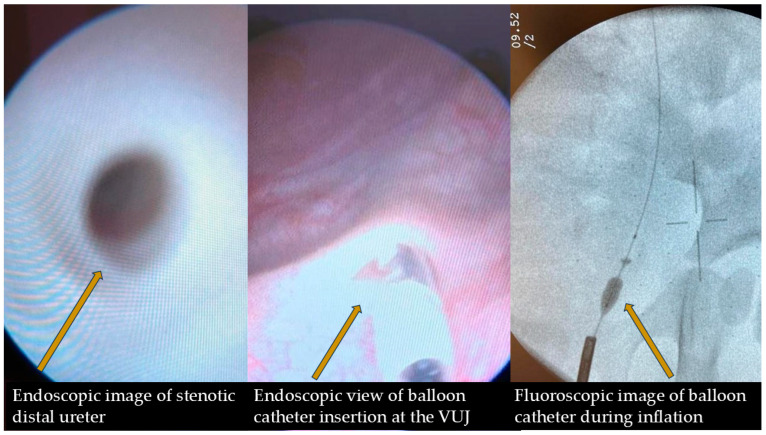
Cystoscopy.

The procedure was performed under general anesthesia using a semi-compliant high-pressure balloon catheter (Marflow-Islikon, Switzerland/Boston Scientific-Marlborough, MA, USA) with a maximum inflated diameter of 4–6 mm and a length of 2, 3, or 4 cm. The choice of equipment was tailored to each patient’s anatomical characteristics, including ureteral size and age. The balloon catheter was introduced retrogradely Via the 5 Fr working channel of a 9.5 Fr Karl Storz cystoscope; this was carried out over a hydrophilic guidewire and advanced up to the ureterovesical junction, where it was maintained. The stenotic area was dilated at a pressure of 7–16 atm for 3 to 7 min in two cycles under endoscopic, ultrasound, or fluoroscopic guidance. Whenever possible, a double-J stent (3–4.8 Fr, Urotech–Rohrdorf, Germany/Marflow-Islikon, Switzerland) was temporarily placed for 4–12 weeks to facilitate urine passage in the postoperative edematous area and prevent restenosis during healing (Figure 3).

### 2.5. Follow-Up and Outcome Measures

Antibiotic prophylaxis was maintained until stent removal. Patients were monitored at 1-month to 3-month intervals during the first year and subsequently at 6-month to 12-month intervals. Postoperative follow-up included clinical examinations, urinalysis and urine culture, and serial ultrasounds. Voiding cystourethrography was reserved for cases of recurrent UTIs after stent removal, while renal scintigraphy was performed in cases of unfavorable imaging outcomes.

Success was defined as symptom remission, improvements in hydronephrosis and ureteral dimensions (assessed by imaging), and the preservation of or improvements in renal function.

Complications such as UTIs, hematuria, pain, restenosis, and stent migration were documented and treated accordingly.

### 2.6. Clavien–Dindo Classification

For the assessment of postoperative complications, the Clavien–Dindo classification was used. This standardized system categorizes complications based on their severity, ranging from minor deviations that require no intervention to life-threatening conditions requiring intensive care or redo surgery. By applying this classification, we ensured an objective and systematic evaluation of postoperative outcomes [20].

### 2.7. Statistical Analysis

Spearman’s correlation analysis was employed to assess the strength and direction of monotonic relationships between variables, while Fisher’s exact test was utilized to determine the statistical significance of associations in categorical data.

## 3. Results

### 3.1. Demographic and Clinical Data (Table 1)

The analysis included five patients (four boys and one girl) and six affected ureters (five on the left and one on the right), resulting in a total of 11 procedures. The median age at the time of surgery was 22 months (range: 2 to 63 months). The follow-up period ranged from 3 months (for more recent intervention) to 12 months (Table 1).

**Table 1:** Results summary.

Patients	5 (4 boys, 1 girl)
Affected ureters	6 (5 left, 1 right)
Total procedures	11
Age at surgery	Median: 22 months (Range: 2–63 months)
Follow-up	3 to 12 months
Detection	80% prenatal, 20% symptomatic (febrile UTIs < 2 years)
Hydronephrosis grades	Grade III: 2 cases Grade IV: 3 cases
Measurements	Ureter diameter: 13 mm Renal pelvis diameter: 19 mm
Surgical indications	Progressive dilation (all cases) Clinical symptoms (3 cases) Documented obstruction (3 cases) Renal function deterioration (1 case)
Surgery details	Duration: 40 min Hospital stay: 2 days Stent duration: ~2 months Stent insertion failed: 18%
Post-op follow-up	3 months to 1 year
Overall Incidence	73% of procedures
Severity Classification	Clavien-Dindo Grade I: 30% Clavien-Dindo Grade II: 20% Clavien-Dindo Grade III B: 50%
Intraoperative Incidents	Balloon rupture (1 case) Stenotic ureteral orifices prevented catheter passage—18%
Postoperative Complications	Stent migration: 28% Febrile UTIs: 18% Transient hematuria: 18% Pain: 9%
Outcome	Re-dilations in 36%—required for ureterovesical junction recalibration. Long-term renal function stable, with gradual hydronephrosis improvement

Prenatal imaging advancements contributed to the early detection of 80% of cases, while the remaining cases were identified symptomatically primarily due to febrile UTIs before the age of two.

Two patients had Grade III hydronephrosis, while three presented with Grade IV. The mean ureteral diameter was 13 mm, and the average renal pelvis diameter measured 19 mm.

No primary surgical intervention was noted, with all cases undergoing surgery after a period of observation.

Surgical indications included progressive ureteral and renal pelvis dilation with parenchymal thinning in all cases, the presence of clinical symptoms in three cases, and documented obstruction in three cases. Additionally, renal function deterioration was observed Via scintigraphy in one case.

The average duration of the surgical procedure was 40 min, with a mean hospital stay of two days. The ureteral stent was maintained for an average of two months. In 18% of the procedures, the insertion of a stent was not possible. Postoperative follow-up ranged from three months to one year after the final intervention (Table 1).

### 3.2. Procedural Success Rate

The procedure was successfully completed in 81.8% of cases, with a final success rate of 83.3% when excluding cases where catheter placement was not feasible. In all successful cases, global renal function was preserved, and longitudinal renal growth remained comparable to the contralateral kidney. Re-interventions accounted for 36% of procedures, primarily for recalibrating the ureterovesical junction (Table 1).

With a *p*-value of 0.571 (>0.05), no significant association was found between antenatal diagnosis and treatment outcomes in this dataset. While early diagnosis may facilitate timely intervention, its impact on treatment outcomes could not be statistically confirmed in this small cohort.

### 3.3. Incidents and Complications (Table 1)

#### 3.3.1. Classification by Severity

Incidents and complications were observed in 73% of procedures, with severity classified as follows: 30% were Clavien–Dindo Grade I, 20% were Grade II, and 50% were Grade III B (Table 1).

#### 3.3.2. Classification by Frequency

Balloon rupture occurred in one case, necessitating procedural resumption due to the unavailability of a replacement balloon catheter. This highlights the importance of the learning curve, proper equipment selection, and ensuring the availability of necessary materials

In 18% of cases, stenotic ureteral orifices prevented catheter passage or balloon stabilization, particularly in younger patients (aged 2 and 5 months), rendering the procedure unfeasible.

Re-dilations were required in 36% of cases for ureterovesical junction recalibration, underscoring the need for careful patient selection and periodic imaging assessments.

Postoperative complications included stent migration in 28% of cases, febrile UTIs in 18%, pain in 9%, and transient hematuria in 18% (Table 1).

Despite these challenges, long-term renal function remained stable, with gradual improvements in hydronephrosis, even in cases requiring additional interventions.

#### 3.3.3. Statistical Analysis of Complications

A moderate negative correlation was observed between patient age at the time of the procedure and the likelihood of complications; however, this result was not statistically significant (*p* > 0.05). Younger patients (under six months) appeared to have a slightly higher risk of complications, but further data are needed to confirm this trend.

Additionally, a moderate positive correlation was found between ureteral diameter and the risk of complications, although this too was not statistically significant (*p* > 0.05). Larger ureteral diameters may slightly increase complication risks.

While trends suggest that younger age and larger ureteral diameters might contribute to higher complication rates, the small sample size limits the statistical power of these findings.

## 4. Discussion

The pathophysiology of primary obstructive megaureter involves a complex interplay of anatomical and functional abnormalities that result in ureteral dilation and urine flow obstruction. This condition is primarily characterized by an abnormally enlarged ureter, which may arise due to intrinsic or extrinsic factors affecting the ureteral wall or surrounding structures. Intrinsic factors include abnormalities in the smooth muscle cells and connective tissue of the ureteral wall, resulting in impaired peristalsis—an essential mechanism for urine propulsion from the kidney to the bladder. Dysplasia or hypoplasia of smooth muscle cells can result in weak or ineffective contractions, causing urine stasis and further dilation. Additionally, excessive fibrosis within the connective tissue may further hinder normal peristaltic movement, exacerbating urine transport dysfunction [18].

Histopathological changes in obstructive megaureter reveal significant alterations in smooth muscle cells and the extracellular matrix. A notable increase in smooth muscle cell density is often observed, and this is likely a compensatory response to increased intraluminal pressure and the heightened demand for peristaltic activity. However, disruptions in normal cell turnover, including altered myocyte apoptosis indices, further impair peristaltic coordination [14].

A key pathological feature of chronic obstruction is the development of fibrosis within the ureteral wall. Prolonged obstruction results in excessive fibrous tissue deposition, reducing ureteral elasticity and further impairing function. This fibrosis is often accompanied by chronic inflammation, exacerbating tissue damage and disease progression. In long-standing cases, the extensive remodeling of the ureteral wall can be observed, further compromising its contractile function [21].

The pathophysiological process of POM often begins in utero, with many cases being detected prenatally through ultrasonographic screening. Early prenatal diagnosis is crucial, as it allows for timely monitoring and intervention, potentially preventing severe renal damage. In cases not detected before birth, POM may be diagnosed postnatally due to recurrent urinary tract infections or incidentally during imaging studies performed for unrelated conditions [22].

Advancements in modern ultrasound techniques have significantly improved prenatal detection rates, with approximately 40% of primary obstructive megaureters now identified before birth. As a result, the proportion of symptomatic children diagnosed postnatally has declined to 22–50% [4,11].

The first 11 procedures demonstrated the effectiveness of this minimally invasive approach while highlighting significant technical and postoperative monitoring challenges. The observed incidence and complication rate in this study (73%) is higher compared to the specialized literature, where values range between 10 and 70%, likely due to a limited cohort and learning-curve-specific issues. However, no major complications, such as ureteral perforation or avulsion, nor vesicoureteral reflux were recorded [23,24].

A systematic review of the efficacy and safety of high-pressure balloon dilatation for primary obstructive megaureter in children, published by Aiello in 2022, identified rates of complications associated with this procedure; these rates ranged from 0% to 50%. Most complications were either infectious in nature or related to the stent’s placement. Some studies reported a high infectious morbidity rate associated with double-J stenting, with rates as high as 70%. It was highlighted that the use of a DJ stent was linked to a significantly higher rate of postoperative complications compared to cases without stenting. The complication rates were 56% in the DJ group versus 15% in the no-DJ group for this study [25].

In our cohort, urinary tract infections were documented only in the presence of the ureteral stent, with no recurrence after its removal. Higher UTI rates in the presence of double-J stents are well documented, with reports ranging from 40 to 70%. Studies suggest that eliminating stenting could reduce infectious morbidity [25,26]. For example, a study comparing patients with and without DJ stents after high-pressure balloon dilations found a higher complication rate in those with stents (56%) compared to those without (15%) [27]. This significant difference highlights the potential drawbacks of stent usage despite its benefits in certain clinical scenarios.

Stent migration, both proximal and distal, is another notable issue. Proximal migration into the ureter and distal migration into the bladder can occur, resulting in additional interventions. In one study of 57 patients treated over a 12-year period, proximal stent migration was observed in four cases, while distal migration was noted in three cases. These migrations can cause discomfort and obstructive symptoms, and this may necessitate further surgical procedures to correct the stent’s position [28].

Endourological approaches are generally associated with fewer severe complications due to reduced tissue trauma and shorter recovery times. Major complications such as ureteral perforation, avulsion, or significant blood loss are rare in endoscopic techniques.

Balloon rupture is a learning curve-specific issue that is rarely reported in the international literature. Restenosis may be classified as a complication in the context of endourological procedures when this occurs as an unintended outcome directly related to the technical aspects of the intervention or the healing process. It may result from excessive scar tissue formation at the site of dilation or surgical repair. Postoperative edema or inadequate stent support during healing can also contribute to restenosis.

These findings can contribute to adjusting therapeutic strategies and standardizing operative protocols, considering aspects related to the learning curve and the management of common complications. The use of standardized protocols—with well-defined pressure regimes, gradual dilation under careful pressure monitoring, appropriate stent selection considering ureteral tortuosity, adequate antibiotic prophylaxis, compliance monitoring, and regular urine cultures to detect potential urinary tract infections early—is a solution—for reducing complications in the endourological treatment of primary obstructive megaureter.

The primary limitations of this study are the small sample size and short follow-up period, which may not adequately capture long-term outcomes, such as restenosis rates. This study represents a preliminary experience with endoscopic balloon dilation for primary obstructive megaureter in a pediatric cohort. Future research with larger cohorts and extended follow-up periods is needed.

The small sample size reflects the rarity of this pathology, particularly in a single-center study. Given the specialized nature of the condition and the strict inclusion criteria, assembling a larger cohort is inherently challenging—an issue commonly encountered in studies on rare congenital anomalies.

However, this limitation is explained by the study’s specific focus on procedural complications, with a total of 11 procedures analyzed. Rather than assessing overall treatment success across a broad population, this study aimed to analyze complications in detail, providing valuable insights into the safety and technical challenges of endoscopic balloon dilation for primary obstructive megaureter in a pediatric cohort.

The follow-up period was sufficient for assessing immediate postoperative outcomes, such as complications. However, evaluating long-term outcomes, including restenosis and renal function deterioration, requires significantly longer follow-up durations. The follow-up period reported in the study refers to the time elapsed since the last procedure. Additionally, we emphasize that this study presents a preliminary experience, and the patients are still being monitored beyond the stated follow-up period.

Multi-center collaborations could address the challenge of small sample sizes by pooling data from multiple institutions, thereby increasing cohort size and enhancing the generalizability of findings. Additionally, prospective studies with structured long-term follow-up protocols could provide a more comprehensive assessment of outcomes, particularly restenosis rates and late renal function changes.

## 5. Conclusions

Endourological techniques offer a viable, minimally invasive option for treating primary obstructive megaureter in children, but they pose technical challenges and a distinct complication profile. Stent migration and restenosis are common, highlighting the need for strict postoperative monitoring and careful patient selection. A well-defined learning curve and standardized protocols are crucial for optimizing outcomes.

This study reported a higher complication rate (73%) than the literature, and this is likely due to its small sample size and short follow-up, although the success rate (83%) remains comparable. However, long-term durability and restenosis risk remain uncertain without extended follow-up studies.

The study’s main limitation is its short follow-up, restricting the assessment of long-term outcomes such as restenosis and renal function progression. While immediate postoperative results were captured, prolonged monitoring is needed to confirm the intervention’s efficacy.

## Data Availability

The original contributions presented in this study are included in the article. Further inquiries can be directed to the corresponding author.

## Abbreviations

The following abbreviations are used in this manuscript:

POM	Primary obstructive megaureter
HPEBD	High-pressure endoscopic balloon dilation
DJ	Double J
UTI	Urinary tract infection
BAPU	British Association of Paediatric Urologists
MRI	Magnetic resonance imaging
CT	Computed tomography
MRU	Magnetic resonance urography
RUS	Renal ultrasound
DRF	Differential renal function
DTPA	Diethylenetriaminepentaacetate
VCUG	Voiding cystourethrography
SFU	Society of Fetal Urology
